# Nasopharyngeal swab or clinical-radiological evidence: the dark side of the moon for cancer patients in the COVID-19 era

**DOI:** 10.2217/fon-2020-0372

**Published:** 2020-05-26

**Authors:** Antonello Veccia, Stefania Kinspergher, Mariachiara Dipasquale, Orazio Caffo

**Affiliations:** ^1^Medical Oncology Department, Santa Chiara Hospital, Largo Medaglie d’Oro 1, Trento, 38122, Italy

**Keywords:** clinical diagnosis, COVID-19, lung cancer, nasopharyngeal swab

Coronavirus disease 2019 (COVID-19) has become a serious public health problem worldwide [1]. It is caused by severe acute respiratory syndrome coronavirus 2 (SARS-CoV-2), but little is known about its mechanisms of action or possible treatments [2]*.*

SARS-CoV-2 positivity is currently confirmed by means of a nasopharyngeal swab, but there is increasing evidence that clinical manifestations of the disease may appear even when a swab is negative, and the presence of the disease may not be demonstrated if the patient dies before it is diagnosed. Cancer patients with COVID-19 have a poorer prognosis than patients without cancer [3], and oncologists frequently observe the dramatic clinical course of the disease in patients without proven infection.

All these issues emerged from a clinical case we have recently observed. A 73-year-old male patient receiving an immune checkpoint inhibitor as first line treatment for metastatic non-small-cell lung cancer was hospitalized due to asthenia, fever, dry cough and dyspnea. Nevertheless, the nasopharyngeal swab was negative. Laboratory tests showed hypoxemia, lymphopenia with normal leukocytes, acute renal failure and increased inflammation indexes. Multiple frosted glass thickenings compatible with bilateral interstitial pneumonitis were described at the chest CT scan. Despite the intensive cares, the patient died of acute respiratory failure 3 days after being hospitalized and after a second negative nasopharyngeal swab.

The clinical history, symptom onset and dramatic disease course of this patient clearly suggested the presence of SARS-CoV-2 infection. First of all, like all cancer patients, he was at higher risk of developing the disease: a recent study of 18 cancer patients, including six with lung cancer, found that they were older, more likely to have a history of smoking, polypnea and more severe computed tomography manifestations, and experienced a significantly shorter time to deterioration than patients without cancer [3]. Second, he was a former smoker with chronic obstructive pulmonary disease, and it has been shown that this clinical condition is an independent risk factor for severe COVID-19 [4]. Third, as a strong inflammatory response can be elicited during immune checkpoint treatment, immunotherapy may increase the risk of COVID-19 with a poorer prognosis [5].

Nevertheless, this diagnosis was not supported by the nasopharyngeal swab results, which should therefore be considered falsely negative. Several factors have been hypothesized to be responsible for the high rate of falsely negative swabs, that represents a serious problem when it comes to defining the extent and severity of the COVID-19 pandemic. A factor is the different quality, sensitivity and specificity of the kits used for the real-time polymerase chain reaction (RT-PCR), that is the standard method to detect SARS-CoV-2. Inappropriate collection, transportation and handling of samples may also affect the results of the test. Mutations in the primer and probe target regions of the SARS-CoV-2 genome can compromise the assay performance in the detection of the virus. Moreover, the false negativity may depend on the considered sample type [6]*.* A retrospective analysis of specimens from multiple sites taken from 205 patients has found that nasal swabs have a lower positivity rate (63%) than bronchoalveolar lavage fluid (93%) [7], and it is likely that tests of multiple sites would reduce the number of falsely negative patients.

Therefore, clinical, radiological and laboratory parameters pathognomonic of COVID-19 play a crucial role in the identification of infected patients.

Multiple subsegmental or segmental ground glass opacities and areas of consolidation on chest computed tomography imaging are typical of COVID-19 [8]. A retrospective study including 36 patients with COVID-19 pneumonia, who were examined with both CT scan and RT-PCR at initial presentation, reported a higher sensitivity for chest imaging (97.2 vs 83.3%) [9].

A final consideration comes from the clinical case we considered and concerns the management of cancer patients in the COVID-19 era.

There are a number of specific guidelines for managing cancer patients during the pandemic [10–12], and some recently published suggestions for lung cancer patients [13]. Before starting oncological treatment, a careful evaluation of the risk/benefit ratio should be made, particularly in the case of elderly patients with co-morbidities who are candidates for immune checkpoint inhibitor treatment.

Thus, in conclusion, regardless of exposure to infected subjects, any patient (mainly if affected by cancer) with flu-like symptoms, severe breathing problems, acute renal failure, laboratory tests indicative of infection and radiological signs of pneumonia should be treated as having COVID-19, even when nasopharyngeal swabs are repeatedly negative. This is essential in order to avoid underestimating the number of positive patients who may continue to spread the infection and to improve our ability to identify COVID-19 infection in cancer patients.
